# Generation and characterization of infectious clones of chikungunya virus from an Indian strain as a resource towards chikungunya vaccine research

**DOI:** 10.1016/j.virusres.2025.199571

**Published:** 2025-04-09

**Authors:** Garvita Mathur, Shakuntala Mahilkar, Sujatha Sunil

**Affiliations:** Vector-Borne Diseases Group, International Centre for Genetic Engineering and Biotechnology (ICGEB), New Delhi 110067, India

**Keywords:** Chikungunya virus (CHIKV), Infectious clone (IC), Genetic determinants, Virus pathogenesis

## Abstract

India has witnessed several devastating chikungunya virus (CHIKV) epidemics since 2005. Developing a stable reverse genetic system using Indian CHIKV strains will be paramount to studying the molecular mechanism of CHIKV pathogenesis.

We generated the first infectious clone (IC) of an Indian isolate of CHIKV and described the procedure in the present report. The IC was constructed directly using the isolate's RNA/cDNA and was further characterized after rescuing the virus. The IC exhibited characteristics similar to those of the natural isolate, such as cytopathic effect (CPE), plaque morphology, replication kinetics, and neutralization behavior. Using the same isolate, we also developed a stable IC with a reporter gene (mCherry) between double sub-genomic promoters. We further evaluated the feasibility of using this IC to test neutralization potential of CHIKV sera in 97 samples and observed that CHIKV patients’ samples from India were able to neutralize this IC significantly better than an Asian genotype IC.

With an impetus on vaccine and therapeutics development for CHIKV in recent years, the ICs generated through our study offer a powerful tool for evaluating several aspects of CHIKV pathogenicity and vaccine efficacy.

## Highlights

• Infectious clone of chikungunya virus (CHIKV) using an Indian clinical isolate has been developed.
• The infectious clone was further tagged with a stable mcherry reporter gene.
• The recombinant viruses showed similar characteristics compared to the natural isolate.
• Assessment of the infectious clone for its applicability in evaluating the neutralization potential of clinically confirmed CHIKV patients’ sera.

RNA viruses are notorious for incorporating genetic modifications in their genomes due to their RNA-dependent RNA polymerase's lack of proofreading activity (Filomatori et al., 2019). Chikungunya virus (CHIKV) is one such RNA virus belonging to the family alphavirus and has re-emerged since 2005, causing huge outbreaks across the globe (The Translational Research Consortia, 2021). Initially categorized into three distinct genotypes based on their geographical presence, post the 2005 pandemic, several lineages of the virus have arisen owing to the positive selection of specific mutations across the virus genome that allowed distinct adaptions in the vector and the host (Chen et al., 2021). With each outbreak, sequence analyses of the circulating viral genome reveal region-specific emergence of sub-lineages attributing to the transmission efficiency of the vectors (Tsetsarkin and Weaver, 2011) as well as the severity and extent of these outbreaks (Jain et al., 2020; Chaudhary et al., 2021). In fact, the spread of CHIKV sub-lineages can be attributed to the virus acquiring secondary adaptive mutations, resulting in the emergence of new variants and reintroduction of these sub-lineages into new geographical regions, resulting in outbreaks as well as altering its vector preference (Tsetsarkin and Weaver, 2011; Tsetsarkin et al.*,* 2014 ; Chaudhary et al., 2021).

CHIKV remains a major public health concern in India. Studies on the circulating Indian strains of CHIKV have provided insights into its evolution, expansion, immunobiology, and outbreak characteristics of the disease (Shrinet et al., 2012; Jain et al., 2017; Verma A et al., 2021, 2020). Owing to the distinct genetic makeup of the circulating CHIKV viruses and the constant reintroduction of the viruses into the country, information currently available regarding genetic determinants suggests that these may contribute to outbreak severity and disease outcome (Jain et al., 2020; Chaudhary et al., 2021).

One important tool for understanding CHIKV pathogenicity is the utilization of reverse genetic systems to characterize the functional attributes of mutations in detail. Several CHIKV cDNA clones have been previously generated and have been used to study different aspects of CHIKV biology, ranging from dual-infection, vector competence, and characterization of viral genes (Xie et al., 2020; Kümmerer et al., 2012; Tsetsarkin et al., 2006; Tsetsarkin and Weaver, 2011; Gorchakov et al., 2012; Tsetsarkin and Weaver, 2011; Teo et al., 2013). Studies have also emphasized the criticality of using an appropriate lineage backbone to study adaptive mutations in CHIKV due to the presence of epistatic or synergistic mutations, which could change the impact of these mutations (Chen et al.*,* 2021; Tsetsarkin et al., 2009). In fact, the impact of point mutations such as E1 A226V on host and vector adaptation, synergistic effect along with E2-L210Q and E2-K252Q especially in the backbone of IOL genotype has underscored the importance of the utility of such reverse genetic systems (Ning et al.*,* 2024).

As a first step towards understanding the relevance of mutations identified in Indian circulating strains and developing a reverse genetic tool to evaluate vaccine and drug candidates, we generated a CHIKV infectious clone (IC) using an Indian strain and also incorporated a mCherry tag to this IC to increase its usability in a variety of future applications. We evaluated the IC for its applicability in evaluating the neutralization potential of clinically confirmed CHIKV patient's sera (Babu et al.*,* 2023).

**Fig. 1:**
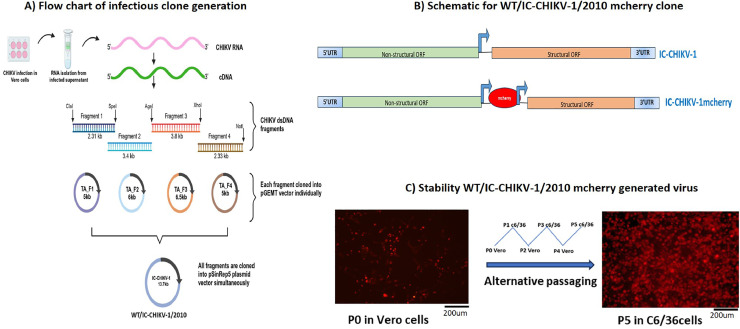
**Generation of infectious clones: (A)** Schematic representation of construction of infectious clone of Indian strain (CHIKV) IND-2010–01 (WT/IC—CHIKV-1/2010). **(B)** Schematic of mcherry gene tagged IC—CHIKV-1(WT/IC—CHIKV-1/2010/mcherry) plasmid between double sub-genomic promoter upstream of structural genes. **(C)** Stability of mcherry gene expression up to five passages after propagation of WT/IC—CHIKV-1/2010/mcherry generated virus alternatively inVero CCL- 81 and C6/36 cell lines. Scale bar, 200 µm.

The full-length IC of CHIKV was generated using an isolate derived from an individual infected during the Delhi 2010 outbreak CHIKV/IND/2010/DEL/01_A (GenBank, accession number: PQ673866) updated version of originally submitted CHIKV/IND/2010/DEL/01(GenBank, accession number: MH124570.1). This clinical isolate has been previously sequenced, identified to belong to the ECSA genotype (Shrinet et al., 2012) and further characterized (Jain et al.*,* 2020). For constructing the IC referred to as WT/IC—CHIKV-1/2010, plasmid pSinRep5 (Invitrogen) was used as a backbone and involved the following steps. Vero CCL-81 cells were first infected with the clinical isolate CHIKV/IND/2010/DEL/01_A, and after 72 hours post-infection (hpi), RNA was isolated from the supernatant of the infected Vero CCL-81 cells. Four overlapping cDNA fragments were then synthesized from CHIKV/IND/2010/DEL/01_A RNA using reverse transcriptase enzyme (Thermo cDNA Synthesis Kit) and amplified using polymerase chain reaction (Thermo Phusion PCR Kit) **(Supplementary Table 1)**. All four PCR fragments were individually cloned into the pGEMT vector and then cloned tandemly in the pSinRep5 plasmid backbone. The Sp6 promoter sequence was introduced upstream of the 5′ end of the CHIKV cDNA sequence, and the viral poly A_40_ tail and *Not*I linearization site were added to the 3′ end (Fig. 1**A**). Introduction of unwanted mutations owing to PCR was routinely checked through sequencing. Eight synonymous mutations and three non-synonymous (nS) mutations were identified during the cloning procedure. The nS mutations were corrected using site-directed mutagenesis (SDM) and confirmed through sequencing **(Supplementary Table 2)**. The finally confirmed WT/IC—CHIKV-1/2010 plasmid was further linearized by the *Not*I enzyme, and the whole genome RNA was synthesized from the sp6 promoter via *in vitro* transcription method (mMESSAGE mMACHINE® Kit, Invitrogen). The *in vitro* transcribed RNA (1μg/ml) was then transfected in Vero CCL- 81 cells using Lipofectamine 3000 (Invitrogen). 72 hours post-transfection (hpt), the specific infectivity of the rescued virus was 2.3 × 10^6^ pfu/μg. To expand the utility of the WT/IC—CHIKV-1/2010 IC, the mcherry reporter gene was incorporated between double sub-genomic promoters to increase the reporter gene's stability (Fig. 1**B**) (Vanlandingham et al., 2005). RNA (1μg) from the tagged infectious clone (WT/IC—CHIKV-1/2010/ mcherry) was transfected in Vero CCL-81 cells, and at 72hpt, the tagged virus was rescued at a specific infectivity of 3 × 10^6^pfu/μg. To further confirm the stability of the expression of mcherry protein, the tagged virus was alternatively passaged in Vero CCL-81 and C6/36 cell lines up to seven passages and evaluated by RT-PCR, sequencing and fluorescence imaging. The mcherry gene was found to be stably expressed for up to five passages alternatively in both cell lines, as shown in (Fig. 1**C**). We believe that this stability will allow the tagged virus to serve as an important tool for studying CHIKV pathogenicity, including dual-fluorescence experiments, as suggested by earlier reports (Kümmerer et al.*,* 2012).

**Fig. 2:**
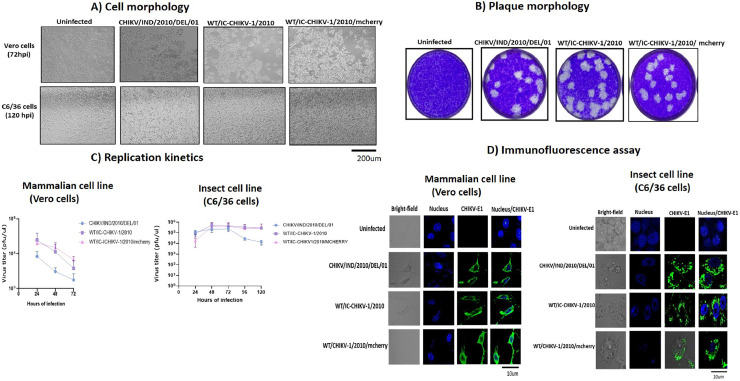
**Characterization of infectious clone-generated viruses against wild-type virus**: **(A)** WT/IC—CHIKV-1/2010 and WT/IC—CHIKV-1/2010/mcherry generated *in vitro* transcripts transfected in Vero CCL- 81 cells and cytopathic effect compared with WT/CHIKV-1/2010 at 72hpi. Scale bar, 200 µm. **(B)** The plaque morphology shows similar medium-sized homogenous plaques after propagation in Vero CCL- 81 cells at 72hpi. **(C)** Replication Kinetics shows a virus propagation pattern with time in mammalian and insect cell lines at MOI-0.1. **(D)** Immunofluorescence assay showing the distribution and expression of CHIKV E1 protein in infected Vero CCL- 81 and c6/36 cells at 24 h and 72 h at MOI-0.1. Uninfected cells were used as controls. Nuclei are stained with DAPI (blue) and CHIKV E1 protein with Alexa Fluor 488 (green). Scale bar, 10 µm. Each experiment was performed in three independent biological replicates with a minimum of three technical replicates. Error bars represent mean±sd.

Upon confirming the ICs, the WT/IC—CHIKV-1/2010 and WT/IC—CHIKV-1/2010/mcherry generated viruses were further characterized and compared with wild-type CHIKV/IND/2010/DEL/01_A at different aspects. The rescued IC-generated viruses were further propagated in Vero CCL-81 and C6/36 cell lines at the multiplicity of infection (MOI) 0.1, which was calculated based on the plaque titer and the infected supernatant collected at different time points. Upon comparing the changes in cell morphology in both cell lines, we observed a similar cytopathic effect (CPE) in terms of cell death at 72hpi in Vero CCL-81 cells for both IC-generated viruses compared to the natural isolate. On the other hand, no prominent CPE was observed in the C6/36 cells even after 120hpi for both ICs and natural isolate virus. The rescued IC viruses also showed homogenous medium-size plaque morphology compared to natural isolate (Figs. 2**A** and 2**B**). The pattern of replication kinetics of IC-generated viruses was compared in both mammalian (Vero) and insect (C6/36) cell lines at different time points at MOI-0.1. The growth kinetics of both IC-generated viruses showed peak viral titer at 24hpi in Vero CCL-81 cells and peak viral titer at 72hpi in C6/36 cells compared to the wild-type virus (Fig. 2**C**). Immunofluorescence assay also showed similar expression of E1 protein (Alexa fluor 488 dye) surrounding nucleus (DAPI) in Vero CCL- 81 cells at 24hpi and in C6/36 cells at 72hpi by both IC-generated viruses compared to the wild-type virus (Fig. 2**D**). Overall, the results suggested that both IC-generated viruses have similar characteristics to natural isolate CHIKV, and incorporating the reporter gene did not change the characteristics of WT/IC—CHIKV-1/2010 generated virus.

Vaccine development for CHIKV has made significant progress over recent years, driven by the global public health impact of the virus. However, the significant success of any vaccine is based on its neuralization against heterologous lineages, as novel CHIKV strains have rapidly emerged during extensive outbreak scenarios. This is particularly relevant considering that most vaccine candidates in advanced stages of development are based on sequences from different lineages. (de Souza et al., 2024; Montalvo Zurbia-Flores et al., 2023).

**Fig. 3:**
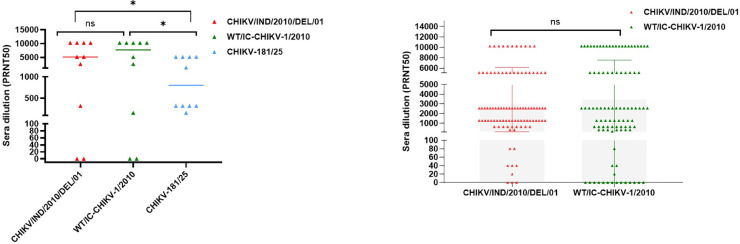
**Infectious clone as a tool to detect neutralization capacity clinical sera samples. (A)** Plaque reduction neutralization assay (PRNT50) comparing the neutralization capacity of 10 clinical sera samples collected after 2016 in India using CHIKV/IND/2010/DEL/01_A, WT/IC—CHIKV-1/2010 and CHIKV-181/25 army clone generated viruses sera. Neuralization of both CHIK/IND/2010/DEL/01 and WT/IC—CHIKV-1/2010 viruses are similar, possibly due to similar lineage backbone (ECSA genotype) compared to the CHIKV-181/25–7097 virus with different lineage backbone (Asian genotype). A comparison of the neutralizing capacity of sera samples using a *t*-test against CHIKV/IND/2010/DEL/01 vs WT/IC—CHIKV-1/2010 showed no significant difference, showing similarity between these two viruses and significant differences (p 0.0284) in neutralization against CHIKV/IND/2010/DEL/01 or WT/IC—CHIKV-1/2010 vs. CHIKV-181/25 army clone-7097 showing differences in the viruses. **(B)** Comparison of neutralization of CHIKV/IND/2010/DEL/01_A and WT/IC—CHIKV-1/2010 viruses using 97 clinical sera samples (PRNT50) from India between 2016–2022. 94 samples (96.90 %) could neutralize CHIKV/IND/2010/DEL/01_A, and 83 (85.56 %) could neutralize WT/IC—CHIKV-1/2010 viruses**.** There is no significant difference between the neutralization of sera samples using both CHIKV/IND/2010/DEL/01 and WT/IC—CHIKV-1/2010, based on the *t*-test, implying both viruses are similar.

As reported previously, the neutralizing antibodies are generated mainly against the E2 epitope and hence, it is essential to test the efficacy of vaccine candidates against the CHIKV lineages circulating in the regions endemic to the virus as adaptive mutations especially in E2 region pertaining to these CHIKV strains could change the binding and neutralization effect of these candidates (Weiss et al.*,* 2020; Verma et al., 2021). In order to be relevant in the CHIKV vaccine and therapeutic research field, we tested our newly constructed Indian IC (WT/IC—CHIKV-1/2010) with ECSA backbone to evaluate the neutralization potential of CHIKV patient sera (Babu et al., 2023). We compared the plaque reduction neutralization test 50 (PRNT 50) results of the IC (WT/IC—CHIKV-1/2010) with the original virus (CHIKV/IND/2010/DEL/01_A) and an infectious clone generated from the Asian genotype, CHIKV-181/25 army clone-7097 (Plante et al., 2011). Results showed that both (CHIKV/IND/2010/DEL/01_A) and (WT/IC—CHIKV-1/2010) exhibited similar neutralization patterns with the clinical sera, whereas the clinical sera displayed decreased neutralizing potential against CHIKV-181/25 (Fig. 3**A**), suggesting that the neutralizing antibodies developed in the Indian scenario could better neutralize the ECSA lineage as compared to the Asian lineage CHIKV and the choice of virus to evaluate neutralizing potential of samples is critical. Our results confirm the findings of earlier studies that emphasize on the differences between the Asian and ECSA lineage on the binding and neutralization characteristics of human sera infected with these genotypes, mainly owing to specific amino acid differences in the E1 and E2 glycoproteins, such as E1-E211K, E2-I2T, E2-H5N, E2-G118S, and E2-S194G (Chua CL et al., 2016). Our strain exhibits some of the above mutations in addition to a few others, whose role in neutralizing potential warrants further analysis (Jain et al.*,* 2020; Chaudhary et al.*,* 2021). To further compare the neutralizing potential of the virus derived from our molecular clone, we evaluated the PRNT50 of 97 samples using both the virus and WT/IC—CHIKV-1/2010. The results provided us information regarding two aspects – Of the total of 97 samples, around 82 samples (85 %) were able to neutralize both the viruses; the neutralization potential in several samples was found to be enhanced in WT/IC—CHIKV-1/2010 when compared to the isolate (Fig. 3**B**). These features make the infectious clone an attractive alternative to the circulating virus to test the neutralizing capacity of sera. Overall, the results suggested that the IC generated from the Indian strain is a powerful tool to test the efficacy of any vaccine candidate.

In conclusion, in countries such as India, where the constant reintroduction of the virus is reported and attributed to outbreaks, it becomes imperative to constantly monitor the efficacy of the vaccine candidates and drug candidates that can be most effective (de Souza et al., 2024). Infectious cDNA clones have proven to be essential tools for studying many aspects of the viral life cycle, and molecular clones of various natural isolates have been instrumental in several recent CHIKV studies (Kümmerer et al., 2012; Tsetsarkin et al., 2009). The reverse genetic system developed in this study using the Indian chikungunya strain can be further used to understand the role of genetic determinants in viral pathogenesis and host response, which could prove to be valuable in developing and evaluating vaccine candidates to combat any future CHIKV epidemics.

## Abbreviations

Chikungunya virus (CHIKV), Chikungunya disease (CHIKD), Infectious clone (IC)

## Funding information

The Department of Biotechnology (BT/PR/45,261/COT/142/30/2022) and TRC—National Biopharma Mission (NBM), BIRAC, Government of India (BT/NBM0101/02/18) funds provided to SS supported the project. G.M. was financially supported by the Department of Biotechnology (DBT, Government of India)

## Ethical clearance

Ethical clearances were collected from the participating institutes, and written consents were obtained from all the participants before collecting clinical samples. (Reference: MAHE EC/004/2020, UEC/32/2013–14, ICGEB/IEC/2019/17, version 3, PGIMER PGI/IEC/2019/000011, TNMC IEC/24/2020, and AIIMS Bhubaneswar T/EMF/Micro/19/09).

## CRediT authorship contribution statement

**Garvita Mathur:** Writing – review & editing, Writing – original draft, Visualization, Validation, Software, Methodology, Investigation, Formal analysis, Data curation. **Shakuntala Mahilkar:** Methodology, Formal analysis. **Sujatha Sunil:** Writing – review & editing, Supervision, Resources, Project administration, Funding acquisition, Conceptualization.

## Declaration of competing interest

The authors declare that they have no known competing financial interests or personal relationships that could have appeared to influence the work reported in this paper.

## Data Availability

Data will be made available on request.

## Appendix Supplementary materials

Image, application 1
