# Supplementary material for: Generation and characterization of infectious clones of chikungunya virus from an Indian strain as a resource towards chikungunya vaccine research
Source: Virus Res. 2025 Apr 9;356:199571. doi: 10.1016/j.virusres.2025.199571 (PMC12022680; doi:10.1016/j.virusres.2025.199571)

**Supplementary Table 1**: Primer list for amplification of four fragments of CHIKV/IND/2010/DEL/01_A RNA using RT-PCR.


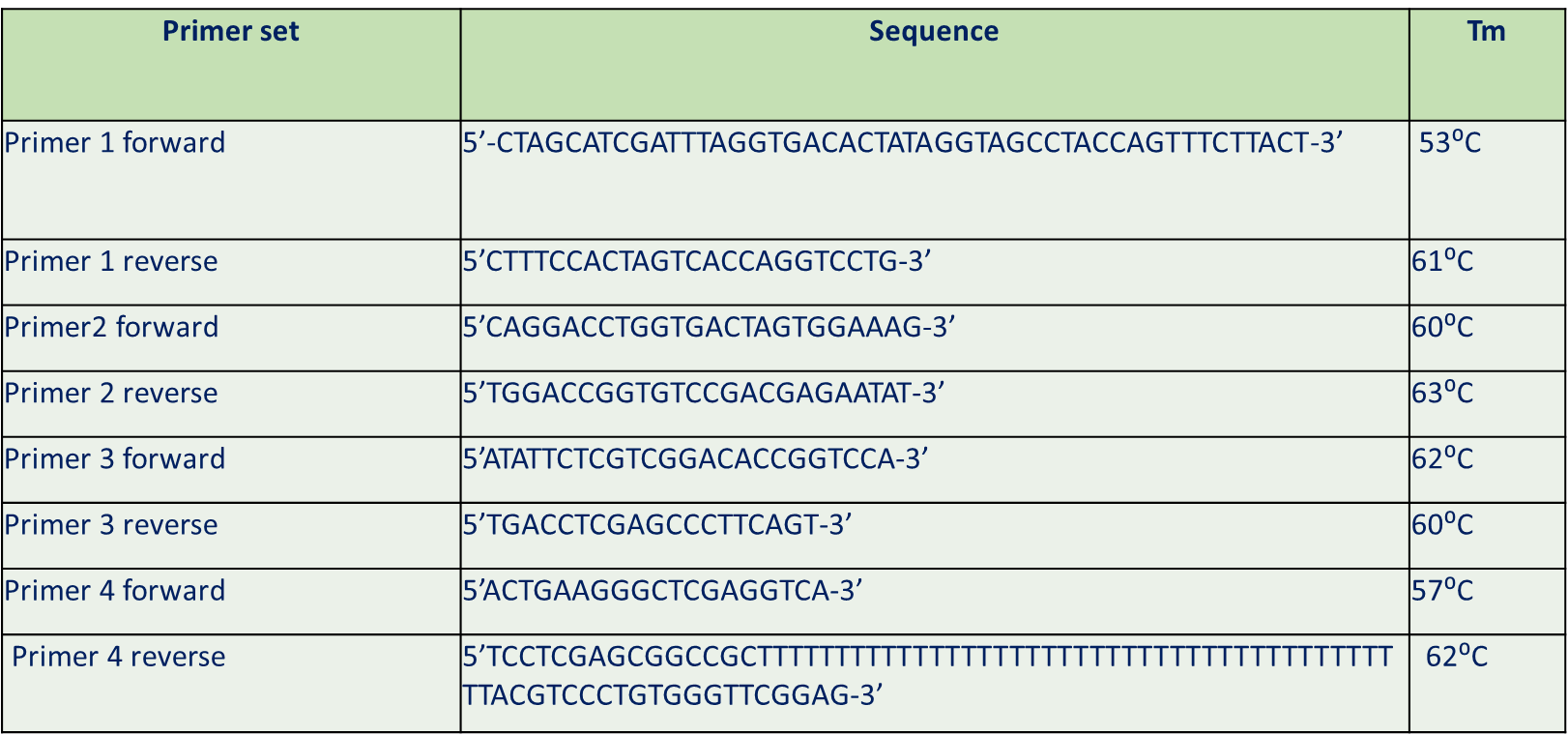


**Supplementary Table 2**: Non-synonymous identified after cloning of WT/IC-CHIKV-1/2010 plasmid and after corrected by site-directed mutagenesis.


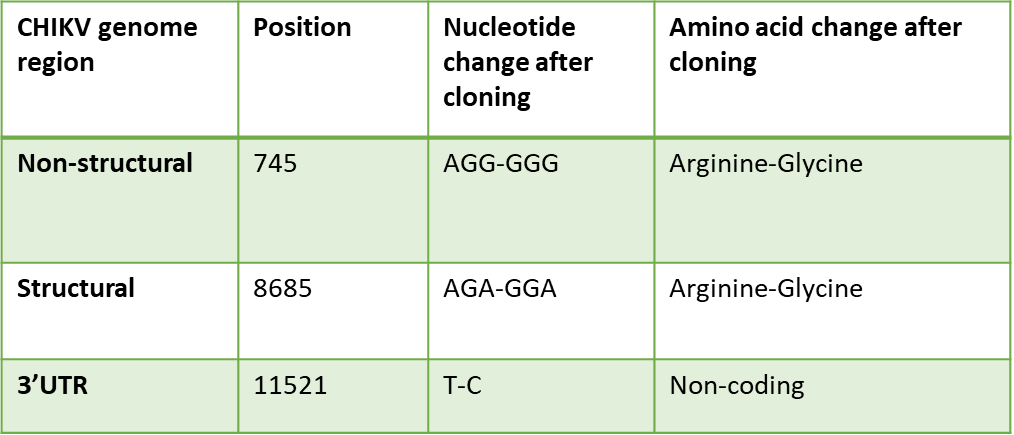

Supplement: Supplementary file 1 [file mmc1.docx]
